# A simplistic approach of algal biofuels production from wastewater using a Hybrid Anaerobic Baffled Reactor and Photobioreactor (HABR-PBR) System

**DOI:** 10.1371/journal.pone.0225458

**Published:** 2019-12-05

**Authors:** Md. Khalekuzzaman, Muhammed Alamgir, Md. Bashirul Islam, Mehedi Hasan

**Affiliations:** Department of Civil Engineering, Khulna University of Engineering & Technology (KUET), Khulna, Bangladesh; Purdue University, UNITED STATES

## Abstract

The current technologies of algal biofuels production and wastewater treatment (e.g., aerobic) process are still in question, due to the significant amount of fresh water and nutrients requirements for microalgae cultivation, and negative energy balance in both processes, especially when considered in the context of developing counties around the world. In this research, a simplistic sustainable approach of algal biofuels production from wastewater was proposed using a Hybrid Anaerobic Baffled Reactor (HABR) and Photobioreactor (PBR) system. The study suggests that the HABR was capable of removing most of the organic and solid (>90% COD and TSS removal) from wastewater, and produced a healthy feedstock (high N: P = 3:1) for microalgae cultivation in PBRs for biofuels production. A co-culture of *Chlorella vulgaris*, *Chlorella sorokiniana*, and *Scenedesmus simris002* showed high lipid content up to 44.1%; and the dominant FAMEs composition (C16-C18) of 87.9% in produced biofuels. Perhaps, this proposed low-cost technological approach (e.g., HABR-PBR system) would connect the currently broken link of sustainable bioenergy generation and wastewater treatment pathway for developing countries.

## Introduction

Currently, the world is facing a global sanitation crisis concerning wastewater management, about 70% of wastewater is treated in high-income countries, 38% in upper-middle-income, 28% in lower-middle-income, and only 8% in low-income countries [1]. On the contrary, most of these developing (low-income and lower-middle-income) counties are located either in a tropical or subtropical region with a warm climate (15–35°C), which is favorable for biological wastewater treatment. Moreover, most of these countries also have electricity scarcity, which makes it challenging to promote aerobic wastewater treatment options [2]. At the same time, there is a global energy crisis due to the rapid utilization of fossil fuels in recent years for industrialization and urbanization. Renewable bioenergy is viewed as one of the ways to improve the current global energy crisis [3]. The energy demand and wastewater pollution situations might be the major limiting factors for the sustainable economic growth of these developing counties in the coming years.

The potential of algae-based biofuels, third-generation bioenergy, is ten thousand (10,000+) times higher than second-generation biofuels, like cellulose-based ethanol and biodiesel (e.g., corn, soybean, oil palm, etc.) [4]. The primary requirements for growing microalgae are sunlight, water, nutrients, and CO_2_. Notwithstanding having the above advantages, the current technologies for algal biofuels generation are still in question. As reported by the National Research Council of the US National Academies, the large-scale production of biofuels from algae is unsuitable using existing technologies due to a significant amount of fresh water, energy and nutrients requirements to grow and harvest enough algae; moreover, using current techniques up to 3,700 liters of fresh water is needed to produce the algal biofuels equivalent to 1 liter of gasoline/fuel[5]. The energy balance for fuel produced from microalgae looks promising, despite contradictions between many studies [6,7].

On the other hand, wastewater is a low-cost and freely available excellent medium for various microalgal growth. It contains macro- and micro-nutrients that support algal growth. The highest microalgae productivity per day reported to date 0.64–14.80 g/L/d of various microalgae species cultivated in different types of wastewater; however, this heterotrophic cultivation can result in high algal biomass production with high lipid content [8]. Whereas, this mode of cultivation possesses limitations, such as requirements of specific heterotrophic algal species, potential algal contamination, and inhibition of algal growth, etc. [9]. Also, various pretreatment methods (such as filtration, autoclaving, UV application, and dilution) are needed for wastewater before using microalgae cultivation, which involves process complexity and cost.

Over the last few decades, anaerobic wastewater treatment technology has become widely adopted owing to its advantages of energy-saving, biogas recovery, and lower sludge production [10,11]. Nevertheless, one of the significant drawbacks of anaerobic wastewater treatment systems is the presence of high nutrients (N and P) in the treated wastewater[12]. This inherent constraints of anaerobic treatment can be dealt with in a complementary fashion to cultivate microalgae for biofuels production[6]. This innovative approach will make the wastewater treatment plant (WWTP) as bio-refinery instead of just a wastewater treatment facility.

Anaerobic baffled reactor (ABR), a third-generation high-rate anaerobic reactor, is highly popular for wastewater treatment because of having significant advantages of low maintenance requirements, rapid biodegradation, low stable sludge yields, excellent process stability on shock loads (e.g. organic and hydraulic), simple and inexpensive construction, and stable operation without requirements for pumping and electricity (e.g. energy positive) [6,13,14]. The major drawback of ABR is reported by researchers[12,14,15] about sludge/solid washout, which ultimately affects ABR treatment efficiency, as a consequence, a poor effluent quality. Sludge washout is directly influenced by reactor up-flow velocity. Higher velocity tends more washout, and lower velocity tends to overcome this problem. In order to avoid the washout problem, filter media can be used, which also increases the risk of clogging and/or maintenance. As an alternative, the fluidized bed reactor (more than 90% treatment efficiency) can also be used that also needs energy for pumping wastewater upward [16]. In this research, a hybrid anaerobic baffled reactor (HABR) has been proposed with improved design concepts and principles, which consisted of a front sedimentation chamber, four regular baffled chambers followed by two floated filter media chambers in order to overcome the above-mentioned drawbacks. The recently conducted hydrodynamic study of the proposed HABR configuration has shown that the optimum reactor performance- low dead space (< 10%), excellent hydraulic efficiency (λ > 0.75), and intermediate mixing pattern (Pe > 10)- were achieved using the proposed HABR with more than five chambers [17].

**Fig 1:**
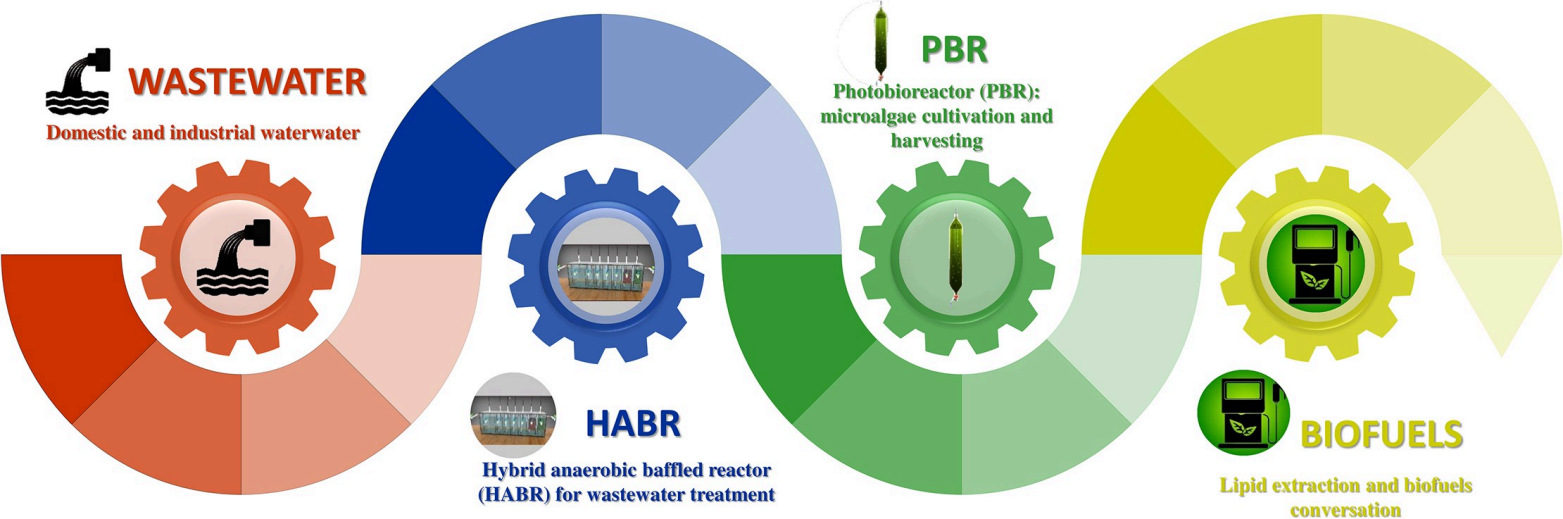
Simplistic Sustainable Approach of Algal Biofuels Production from Wastewater Using a HABR-PBR System.

Although much research has been conducted on microalgae cultivation using raw wastewater or secondary/tertiary treated wastewater [8,9,18–23], few studies have been carried out using anaerobic bioreactor effluent for microalgae cultivation which would be more cost-effective and sustainable for biofuels production and wastewater treatment. Therefore, this research work aims to determine the feasibility of biofuels production using effluent from the proposed HABR followed by microalgae cultivation in a photobioreactor (PBR) in the tropical climate of Bangladesh (e.g., developing country). The main objective of the research was to develop a low-cost, sustainable bioenergy generation corridor and to solve wastewater management/treatment issues (i.e., a problem is a solution or resource) using this HABR-PBR system (Fig 1).

Also, the temperature has a significant effect on reactor treatment efficiency. Researchers [24–26] had shown that treatment efficiencies of the ABR deteriorated significantly when the temperature dropped below 15°C. To overcome temperature effects, decreasing hydraulic retention times (HRT) or heating of wastewater could achieve higher removal efficiency [12], which also involves cost and energy. Therefore, the present study also examined the effect of the insulation of HABR (e.g., HABR (I)) in comparison with uninsulated HABR (e.g., HABR (U)) on effluent quality to cultivate microalgae in PBR for biofuels production.

## Materials and methods

### Wastewater treatment by HABRs

#### HABR configuration and operation

**Fig 2:**
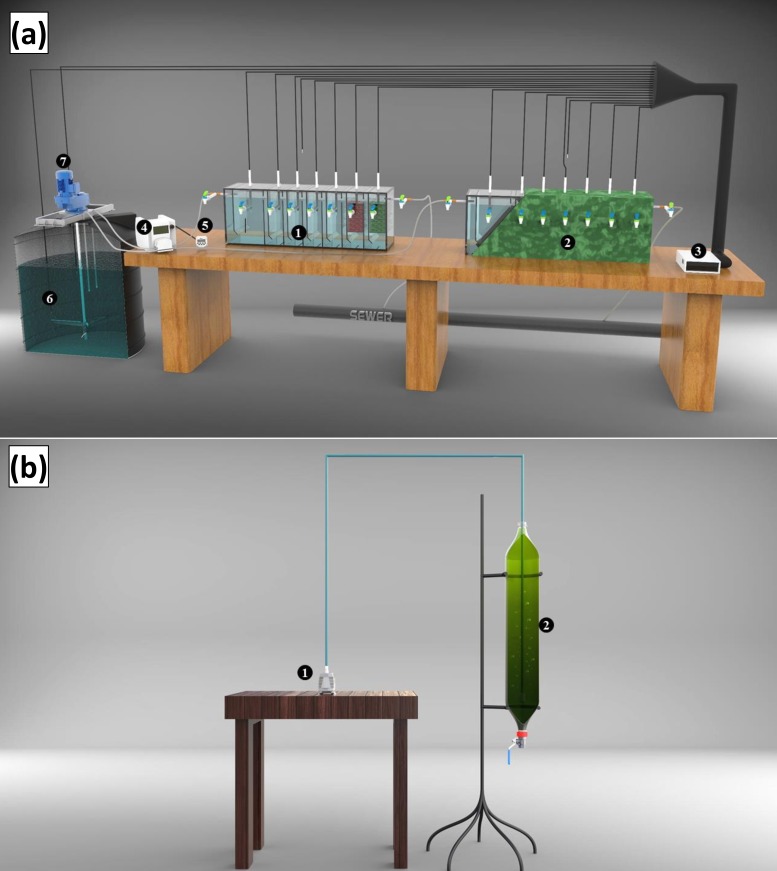
Schematic of the experimental set up of HABR-PBR system. (a) HABR (U) and HABR (I) (1 –uninsulated HABR (U), 2 –insulated HABR (I), 3 –Temperature data logger, 4 –peristaltic pump, 5 –Sino timer, 6 –feed tank, 7 –mixing device). **(b)** Photobioreactor (PBR) setup including gravity settling (1 –air pump, 2 –PBR).

The schematic diagram of the experimental set up with both uninsulated HABR (HABR (U)) and insulated HABR (HABR (I)) is shown in Fig 2(A). Both reactors were identical in the configuration, as summarized in Table 1. These HABRs were constructed using the acrylic sheet with external dimensions of 90, 20, and 30 cm for length, width, and depth, respectively. The effective volume of HABR (U) and HABR (I) were 36.38 L and 36.39 L, respectively. Each HABR consisted of a front sedimentation chamber (U-1 and I-1), four regular chambers (U-2 to U-5, and I-2 to I-5) followed by two floated filter media chambers (U-6 and U-7; and I-6 and I-7). The first chamber volume, designed as the settling chamber, was twice the subsequent chambers. Each chamber was again divided into two portions by hanging baffles, which separated the chamber in down- and up-flow zone. The ratio between down-flow and up-flow was 1:4, and the bottom portion of the baffle was inclined at 45°. The sampling port was installed in the chamber on both HABRs, which was located at 20 cm from the base on the front side of each reactor. Approximately 400 gm of shredded (e.g., grinding to make small pieces) soft drink lid were loosely placed as floated filter media in the last two chambers of each reactor (Table 1). These locally available materials were used due to their favorable physical properties that would act as floated filter media, and won’t let the reactor failure for clogging during wastewater treatment. Polyurethane foam (Pu Foam, Boya, Korea) was used for insulating one HABR by applying a 2-inch liquid foam layer and let it dry at room temperature (21–25°C). Arduino UNIO technology (each chamber equipped with a DS18B20 waterproof digital temperature sensor connected to the data logger) was also installed to monitor temperature (24/7–1 h interval) during operation. Each compartment also had a 3-mm vent pipe (located behind temperature sensors pipe) to exhaust gas (e.g., methane).

**Table 1:** Summary of HABR configuration (identical for uninsulated and insulated).

Design Parameter	Specification
ABR dimensions	90 cm (L) x 20 cm (W) x 30 cm (H)
Effective volume	36.4 L
First chamber/settler	2V (where, V- the volume of the subsequent chamber)
Deflector angle of the hanging baffle	45°
Down-flow/Up-flow	1:4
Type of filter media	Floated filter media (shredded soft drink lid), density-109 kg/m^3^, Specific gravity– 0.93) (grinding of soft drink lid)
Sampling Port	20 cm (from the base) at center
In-let/out-let	In-let (27 cm from base); Out-let (25 cm from base)

Both HABRs were operated under the same ambient conditions to evaluate the effluent quality for microalgae cultivation. Domestic wastewater was collected from KUET (Khulna University of Engineering & Technology, Khulna, Bangladesh) campus residential area, and stored in a feed tank system. The feed tank system consisted of 5 500-L premium grade PVC tanks interconnect each other, one tank (feeder) equipped with mixing device for uniform feed strength. The characteristics of influent wastewater are presented in Table 2. The wastewater was then fed from feeder tank to both HABRs continuously (running system 24/7–10 min feeding in each h) using a peristaltic pump (WT600-1F, Longer pump Co., China). The mixing device in the feeder tank and peristaltic pump were also connected to a Sino-timer (Sino timer, China), which was programmed to run the system for 10 min/hr during feeding time throughout the entire experiment. The HRTs of both HABR (U) and HABR (I) were 30 h for the first 40 days and then 20 h for remaining 100 days, except the system was put idle for 42 days (between 60 and 102 days).

**Table 2:** Characteristics of influent and effluent wastewater, and final removal efficiency of HABR (U) and HABR (I).

Parameter	Unit	Influent concentration		Effluent concentration		Removal efficiency (%)	
		HABR (U)	HABR (I)	HABR (U)	HABR (I)	HABR (U)	HABR (I)
pH	-	8.1±0.2	8.1±0.2	8.0±0.2	8.0±0.1	-	-
ORP	mV	44.2±85.5	42.5±58.5	101.4±75.6	57.2±45.7	-	-
Turbidity	NTU	490±377	563±343	11±7	13±8	98±1	97±2
COD	mg/L	575±239	638±240	39±37	64±50	93±7	89±9
NH_4_^+^-N	mg/L	54.4±20.6	52.3±21.9	37.8±28.0	13.3±29.9	42±36	47±42
NO_3_^-^-N	mg/L	31.1±48.3	31.9±43.6	24.7±34.5	14.9±26.5	63±30	75±26
NO_2_^-^-N	mg/L	15.8±29.4	14.3±27.7	12.1±22.7	9.2±18.7	-	-
PO_4_^3-^	mg/L	25.7±12.6	38.4±21.8	29.3±18.0	17.2±25.3	33±22	38±24
TSS	mg/L	335±258	625±414	15±5	20±17	91±9	96±5
VSS	mg/L	195±148	367±210	9±3	12±8	91±9	95±9
Total coliform^+^	mg/L	5.4x10^5^	3.3 x10^5^	1.4 x10^5^	2.3 x10^5^	75%	25%
Faecal coliform^+^	mg/L	1.9 x10^5^	1.2 x10^5^	0.90 x10^5^	0.95 x10^5^	59%	33%

Note

^+^ - Average of sampling on day 27 and 120

#### HABR inoculation

Both HABRs were inoculated with septic sludge collected from KUET campus residential area. The stable septic sludge was sieved using a 2.0-mm mesh prior to adding into reactors. In each HABR, approximately 9.2 L (3.2 L in chamber 1, and 1.5 L in each chamber 2–5) of sludge was added to chamber 1 to 5, the remaining volume of the chamber being filled with septic tank effluent including chambers 6 and 7. This seeded sludge contributed substantially to the solid requirement in the reactor system after settling. The sieved sludge contained total solids (TS) of 8960±1824 mg/L and total volatile solids (TVS) of 6880±1137 mg/L. After inoculating, both HABRs were left at ambient temperature for 30 d without further modification.

#### Wastewater sampling and analysis

Nine wastewater samples from each HABR (U & I): raw (U-R and I-R), seven sampling ports of each HABR (U-1 to U-7, and I-1 to I-7), and effluent (U-E and I-E), were collected routinely for laboratory analysis. Raw and effluent samples were analyzed for pH, oxygen redox potential (ORP), turbidity, total chemical oxygen demand (COD), ammonia-N (NH_4_^+^-N), nitrate-N (NO_3_^-^-N), nitrite-N (NO_2_^-^-N), Orthophosphate (PO_4_^3-^), total suspended solids (TSS), volatile suspended solids (VSS), total coliform (TC) and fecal coliform (FC) according to the standard methods [27]. Samples collected from reactor chambers were also analyzed for selected parameters.

### Microalgae cultivation, harvesting, lipid extraction, and biofuels conversation

#### PBR configuration

The microalgae cultivation, harvesting, lipid extraction, and biofuels conversion are challenging steps and are on-going research topics throughout the world. In the present study, priority was given on cost-effectiveness (low-cost) in PBR design; hence, the gravity settling was considered for microalgae harvesting after cultivation. Four (4) identical 3.5 L capacity photobioreactors (PBR-1 to PBR-4) were constructed using locally available 2-L clear coke soft drink bottles attached back-to-back and were placed vertically with support for microalgal cultivation. The conical bottom shape of the PBR allowed the microalgae to settle at the bottom for harvesting (Fig 2(B)). A flow control valve was also installed at the lower end of the PBR for the collection of concentrated wet biomass after cultivation.

#### Microalgae strains selection and cultivation

To avoid the major risk of contamination of microalgae monocultures [28], a co-culture of *Chlorella vulgaris*, *Chlorella sorokiniana*, *and Scenedesmus simris002 (ratio = 1*:*2*:*1)* were used in the present study for microalgae cultivation. All three freshwater microalgae have been reported as having high lipid content (14–56.7%) by researches [29–31]; moreover, they also have good tolerance in saline water[31]. The research area (KUET campus) is located in a coastal zone with a high salinity in supplied water, which also contributes to the domestic wastewater.

Four (4) photobioreactors (PBR-1 to PBR-4) were used for microalgae cultivation. Two (2) two-headers (i.e. 2 x 3 L/min) capacity aquarium air pump (Venusaqua, China) were also installed for airflow to supply CO_2_ in PBR during cultivation. Each air pump header was also connected by a 5-mm airflow tube to an air stone. The air stone was placed approximately 4 cm above the bottom of the reactor because of allowing gravity sedimentation of microalgae for harvesting. The airflow system was operated by the above mentioned Sino-timer (Sino timer, China), which was programmed to run the air pumps (e.g., CO_2_ supply) for 10 min/hr (running continuously 24/7) during eight days of cultivation period. These PBRs were placed in different locations of a shed room (with the only roof) to observe the effect of sunlight irradiation on microalgal growth.

When comparing the effluent quality of both reactors regarding nutrients content, it appeared that HABR (U) effluent was higher in nutrient (high N and P) than HABR (I) (Table 2). In addition, the total coliform (TC) and fecal coliform (FC) results showed that the higher TC/FC removal was achieved in HABR (U) (e.g. 75% TC and 59% FC removal in HABR (U)) (Table 2), which reduced the risk of bacterial contamination during microalgae cultivation. Therefore, the effluent of HABR (U) was considered a healthy feedstock (high N: P = 3:1)[32,33] and was used as an influent of PBRs for microalgae cultivation in this study. Besides, selected wastewater parameters pH, DO, EC, ORP, and water temperature; and light irradiation (AM, noon, and PM) were also monitored for each day of cultivation period (8 days).

#### Microalgae growth kinetics and biomass estimation

The microalgal growth kinetics and biomass production potential were assessed for four PBRs, especially for the consideration of the light irradiation pattern on growth rate during cultivation. The optical density of microalgae cell culture suspensions was observed at 680 nm every day during eight days of cultivation using a HACH DR 3900 spectrophotometer. Biomass productivity (g/L/d) was calculated from the variation in biomass concentration (g/L) within a cultivation time (d) according to the following Eq (1) [34,35]:

$$P=\frac{X_{t}-X_{0}}{t_{t}-t_{0}}$$ (1)

Where ‘X_t_’ is the biomass concentration at the time ‘t’, while ‘X_0_’ is the initial biomass concentration at inoculation time t_0_. P_max_ (g/L/d) was designated to the maximum productivity.

The specific growth rate (μ, d^-1^) was calculated using Eq (2):

$$\mu =\frac{{lnX}_{t}-{lnX}_{0}}{t_{t}-t_{0}}$$ (2)

The maximum specific growth rate (μ_max_, d^-1^) was determined from the different μ values calculated, while the maximum biomass obtained was designated as X_max_ (g/L). Cell doubling time (*t_d_*(*μ_avg_*, d) was estimated using Eq (3) [34]:

$$t_{d}(\mu_{avg})=\frac{ln2}{\mu_{avg}}$$ (3)

Carbon dioxide uptake rate ($P_{{CO}_{2}}$) (g/L/d) was measured using Eq (4), whereas ‘P’ is the productivity calculated above in Eq (4)[34].

$$P_{{CO}_{2}}=1.88\;X\;P$$ (4)

#### Microalgae harvesting and lipid extraction

The concentrated wet microalgae were harvested from the bottom of each PBR after cultivation using the flow control valve for analyses. The harvested algal biomass was collected together, and then split into six (6) samples: four (4) samples were used to determine moisture content by keeping samples at 65°C in oven for overnight[36], and then two (2) (microalgae dry cell 1 and microalgae dry cell 2) out of these four (4) samples were taken for Fourier transforms infrared (FTIR) analysis; and two (2) wet microalgal samples were used to determine lipid content (LP1 and LP2), which were then converted to biofuels (biodiesel 1 and biodiesel 2) after the transesterification process[34,36,37].

As mentioned above, two (2) concentrated wet samples were used for lipid extraction using a single-step lipid extraction procedure[36]: 8 ml of a 2:1 chloroform: methanol (v/v) mixture was added to fresh microalgal biomass paste in each centrifuge tube. The biomass was manually suspended by vigorously shaking the tubes for a few seconds, and 2 ml of a 0.73% NaCl water solution was added. Phase separation was facilitated by 2 min of centrifugation at 350 g using NUVE NF 800 centrifuge, and the lower phase was recovered using a micropipette and was placed in an aluminum foil cup for overnight solvent evaporation at room temperature followed by gravimetrical determination of the lipid content (LP1 and LP2).

#### Transesterification process

Methylation of extracted lipids (LP1 and LP2) was conducted for converting all fatty acids to their corresponding methyl esters through the transesterification process, and the profile was then analyzed using FTIR and Gas Chromatography-Flame Ionization Detector (GC-FID) as the discussed in following sections. Also, a vegetable oil sample was also converted to biodiesel (e.g., biodiesel (veg. oil)) through the transesterification process and was analyzed using FTIR for comparison.

Fatty acid methyl esters (FAMEs) were prepared using 30 mg of total lipid dissolved in 1 ml of methanol, was then mixed with 1 ml of 1% NaOH solution prepared in methanol[38]. To this solution, equal volume (i.e., 2 ml) of 5% HCl (13.5 ml concentrated HCl in methanol) solution was added and then heated at 75°C for 15 min. This solution was allowed to cool at room temperature, and 1 ml of distilled water was added and shaken. The organic layer containing FAMEs was carefully transferred to a new clean vial for FTIR and GC-FID analysis [39].

#### Fourier transform infrared spectroscopy (FTIR) analysis

The composition of microalgae dry cells and biofuels, and the type of functional groups of the algal dry cell and biofuels were assessed through FTIR spectroscopy study [40]. FTIR analyses were conducted on microalgae dry cell (1 & 2), biodiesel (veg. oil), and biodiesel (1 & 2) at room temperature using Shimadzu (IRTracer-100) FTIR spectrophotometer [34]. The dried algal biomass samples were further broken into powder. Dried algal cells were pressed against the diamond cell before scanning. The extracts from these samples were observed for their functionalities in the spectrogram. The spectra were collected in the mid-IR range from 4000 to 800 cm^-1^ (at a spectral resolution of 2 cm^-1^), and data were analyzed using Microsoft Excel, irAnalyze-RAMalyze (LabCognition GmbH & Co. KG) and KnowItAll (Bio-Rad Laboratories Inc., Pennsylvania, USA).

#### Gas chromatography-flame ionization detector (GC-FID) analysis

The FAMEs composition of the biodiesel sample (biodiesel 2) was analyzed using gas chromatography (GC-2010 Plus, Shimadzu, Japan) system with a flame ionization detector (FID) and equipped with a capillary column (MEGA SE-52 25 m × 0.25 mm × 0.25μm). 1.0 μL of methyl ester sample solution was injected for FAME analysis. Nitrogen was used as a carrier gas. The injector temperature was 180°C, which was increased to 250°C at a temperature gradient of 15°C/min. The identification of FAMEs was made by comparing the retention times with those of the standard compounds[39].

#### Wastewater analysis after microalgae harvesting

After harvesting the concentrated wet microalgae from the PBRs, the remaining wastewater was analyzed for pH, ORP, Turbidity, COD, NO_3_^-^-N, NO_2_^-^-N, PO_4_^3-^ parameters.

### Statistical analysis

Data analysis was performed with Microsoft Excel. The one-way ANOVA was used to determine the significance of the analytical results and difference between groups, and P<0.05 was considered as significant (S1 File).

## Results and discussion

**Fig 3:**
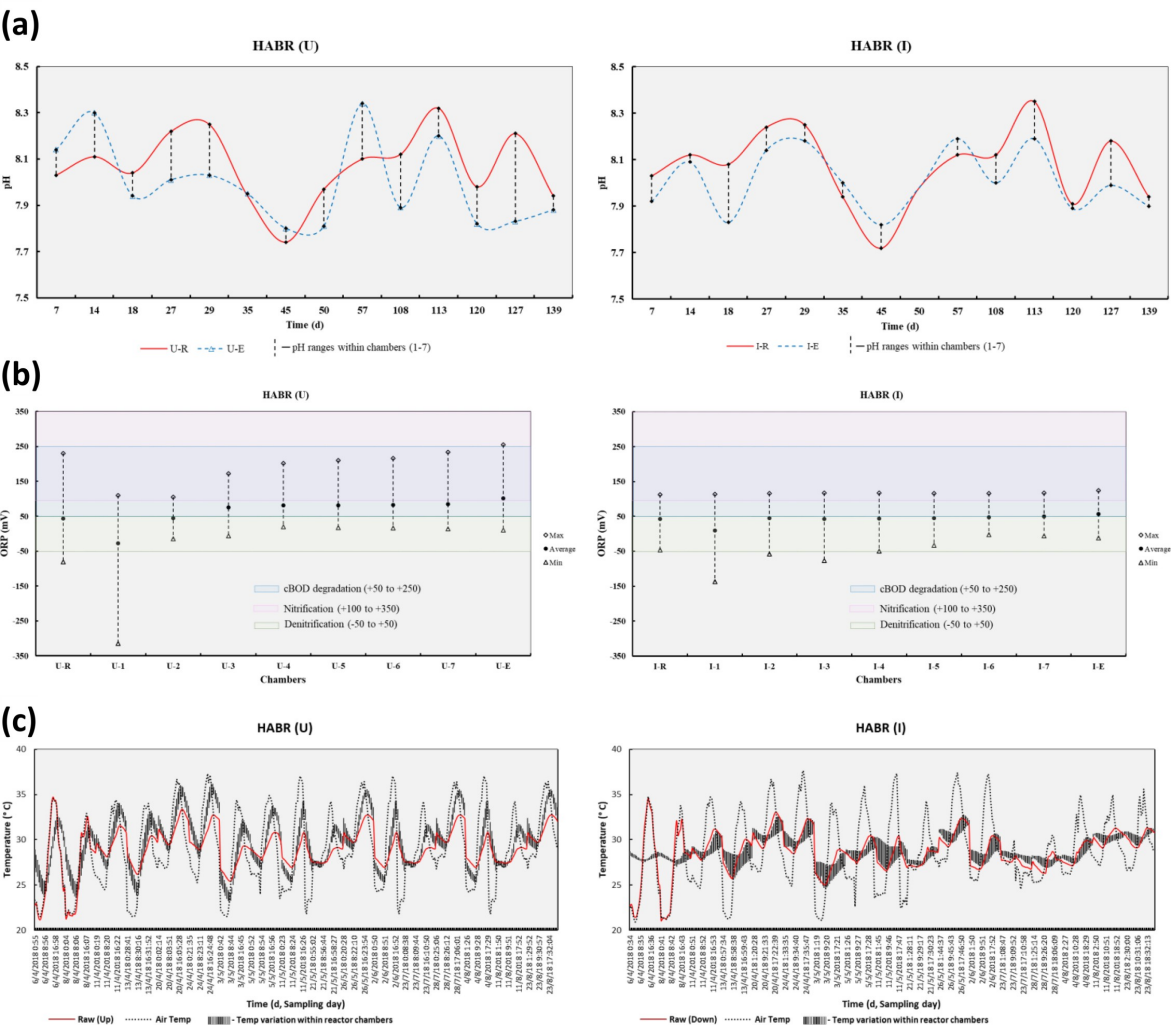
pH, ORP, and Temperature data of both HABR (U) and HABR (I). **(a)** pH and **(b)** ORP of influent, effluent, and chambers of both reactors. **(c)** Temperature data of raw, air, and variation within reactor chambers.

In the present study, pH and ORP were monitored for influent (U-R and I-R), effluent (U-E and I-E), and samples from each chamber of HABRs (U-1 to U-7, and I-1 to I-7) as presented in Table 2 (S1 and S2 Tables), and as shown in Fig 3(A) and 3(B). The pH was 8.1±0.2 for influent wastewater for both HABRs; and ORPs were 44.2±85.5 mV and 42.5±58.5 mV for influent wastewater of HABR (U) and HABR (I), respectively. The ORP ranges from -315 to 255 mV in the chambers of HABR(U), and from -137 to 117 mV in the chambers of HABR (I), respectively. This indicates a favorable anaerobic/anoxic/oxic condition existed in both HABRs for organics biodegradation as well as nitrification/denitrification/anammox processes. The temperature was also monitored using the Arduino UNIO temperature data logger as shown in Fig 3(C) (S2 File). It appeared that the insulation provided a better temperature control in HABR (I) during operation, whereas there was a significant temperature variation observed within the chambers of HABR (U) (e.g., mostly followed influent wastewater and ambient air temperature patterns). This variation of temperature within the reactor’s chambers ultimately affected the treatment efficiency of the HABRs.

### Wastewater treatment by HABRs

#### COD and solids removal

COD, turbidity, TSS, and VSS concentration of influent and effluent; and their removal efficiencies for both HABR (U) and HABR (I) are presented in Table 2. As the actual domestic wastewater was used for the experiments, the influent COD concentrations were observed to be varying [41]. The results indicated overall 93±7% and 89±9% COD removal by HABR (U) and HABR (I), respectively. It appeared that the COD removal efficiencies for both reactors fluctuated (e.g., 79–100% in HABR (U) and 86–100% in HABR (I)) during these experiments. The COD removal was 91±7% in HABR (U) and 86±8% in HABR (I) for 30 h HRT; and 94±7% in HABR (U) and 91±10% in HABR (I) for 20 h HRT, respectively, which indicates the COD removal efficiencies increase with lower HRT (e.g., 20 h). The organic loading rate (OLR) were 0.61±0.30 kgCOD/m^3^.d for HABR (U), and 0.67±0.31 kgCOD/m^3^.d for HABR (I), respectively. Also, the organic removal rate (ORR) were 0.57±0.29 kgCOD/m^3^.d for HABR (U) and 0.61±0.31 kgCOD/m^3^.d for HABR (I). The results indicate the COD removal is directly influenced by OLR [41,42]. No significant influence on COD removal efficiency was observed because of the insulation of the HABR. The average effluent COD was 39±37 mg/L for HABR (U), and 64±50 mg/ for HABR (I). As mentioned above, the ORP data indicated that there were favorable oxic/anoxic/anaerobic conditions existed in both the reactor’s chambers for biological organic matter degradation[43].

During the experiments, turbidity was measured for influent and effluent samples for both reactors (Table 2). The turbidity reduced significantly from 490±377 NTU to 11±7 NTU of in HABR (U), and from 563±343 NTU to 13±8 NTU in HABR (I); resulting 98±1% and 97±2% turbidity reduction in HABR (U) and HABR (I), respectively. Both HABRs showed superior performance for TSS and VSS removal: 91±9% for both TSS and VSS removal in HABR (U), and 96±5% of TSS and 95±9% of VSS in HABR (I). The effluent TSS was 15±5 mg/L and 20±17 mg/L in HABR (U) and HABR (I), respectively. Also, the effluent VSS was 9±3 mg/L and 12±8 mg/L in HABR (U) and HABR (I), respectively, suggests minimum biomass washout from both reactors. Feng et al. [24] have studied a bamboo carrier ABR and reported effluent TSS 14.35±3.01 mg/L (e.g., TSS removal of 81.92±3.53%) when operating at constant temperature 28±1°C for 48 h HRT. In this study, both HABRs suggested higher TSS removal efficiency in comparison with their research. The average VSS/TSS ratio of influent wastewater was 0.55 for HABR (U), and 0.59 for HABR (I) suggested high VSS/TSS ratio for successfully anaerobic digestion [44]. A minor increase (e.g., 4–5%) of TSS or VSS removal was observed in HABR (I) because of insulation, which was perhaps due to ideal intermediate dispersion (e.g., weaker) existed in HABR (I) as suggested from a hydrodynamic study of the HABR [17].

#### Nitrogen and phosphate removal

**Fig 4:**
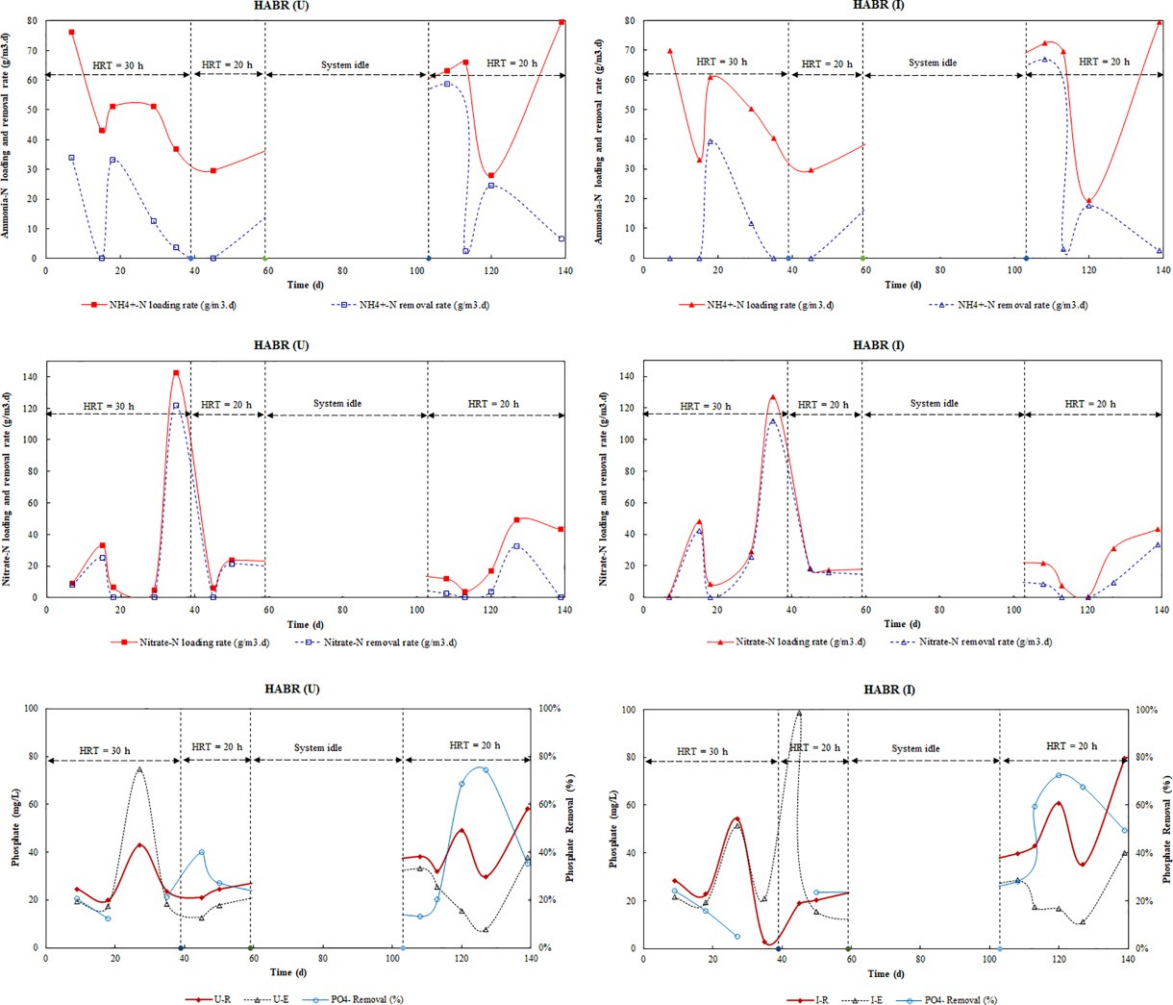
NH_4_^+^-N, NO_3_^-^-N, and PO_4_^3^- loading and removal rate of HABR (U) and HABR (I).

NH_4_^+^-N, NO_3_^-^-N and NO_2_^-^-N concentration of influent and effluent; and NH_4_^+^-N and NO_3_^-^-N removal efficiencies for both HABR (U) and HABR (I) are presented in Table 2 (S3 File). Fig 4 shows NH_4_^+^-N and NO_3_^-^-N loading and removal rate for both HABRs. It appeared that the NH_4_^+^-N removal rate primarily depended on its loading rate in each reactor (significant, p = 0.0007<0.05 for HABR(U); p = 0.009<0.05 for HABR(I)). The average NH_4_^+^-N removal efficiency was 42±36% in HABR (U), and 47±42% in HABR (I). The recent study showed that the insulation of HABR increased NH_4_^+^-N removal by 7%[45], where the both HABRs were operated for 50 d (40 d for 30 h HRT, and 10 d for 20 h HRT). It was also observed that NH_4_^+^-N removal primarily occurred due to nitrification, which was observed high on day 18 and then gradually decreases afterward.

In this study, both reactors were operated for 140 d (40 d for 30 h HRT and remaining 100 d for 20 h HRT, except the system was idle between 60 and 102 d). It appeared that NH_4_^+^-N removal efficiency was higher 44±29% in HABR (I) than 36±24% in HABR (U) at 30 h HRT; however, similar NH_4_^+^-N removal efficiency 48±51% and 48±49% in HABR (I) and HABR (U), respectively, was observed at lower 20 h HRT. On the other hand, when looking at the NH_4_^+^-N loading rate, it was almost similar in both 30 h and 20 h HRT. At 30 h HRT, the NH_4_^+^-N loading rate was 0.05±0.02 kg/m^3^.d in both HABRs. At 20 h HRT, NH_4_^+^-N loading rate was also 0.05±0.02 kg/m^3^.d in both HABRs. The results suggested that at 30 h HRT, based on ORP (+49 to +96 mV in HABR (U), and +49 to +114 mV in HABR (I)), NH_4_^+^-N was primly removed by nitrification because of oxic/anoxic condition existed in both HABRs; however, at low 20 h HRT, NH_4_^+^-N was removed by both nitrification and perhaps anammox processes (-315 to +234 mV ORP in HABR (U), and -137 to +117 mV ORP in HABR (I)).

Fig 4 also shows that the NO_3_^-^-N removal rate followed its loading rate. An average NO_3_^-^-N removal efficiency due to denitrification was 63±30% in HABR (U), and 75±26% in HABR (I). It appeared that NO_3_^-^-N removal rate was not depended on NO_3_^-^-N loading rate for both HABRs (not significant, p = 0.46>0.05 for HABR(U); p = 0.60>0.05 for HABR(I)). However, it was actually depended on NH_4_^+^-N loading rate (significant, p = 0.04<0.05 for HABR(U); p = 0.03<0.05 for HAB(I)). The influent ORP ranges (-80 to +230 mV in HABR (U), and -46 to +112 mV in HABR (I)) suggested that oxic/anoxic favorable condition for nitrification and denitrification [46]. However, these processes were not stable because of the significant variation of NH_4_^+^-N and NO_3_^-^-N concentration in the raw wastewater.

Phosphate (as Orthophosphate, PO_4_^3-^) concentration of influent and effluent wastewater and their removal percentages are shown in Fig 4 (S3 File). An average PO_4_^3-^ removal efficiency was 33±22% in HABR (U), and 38±24% in HABR (I). The results showed unstable phosphate removal in both reactors, Kishida et al.[46] have reported similar findings from their study. The recent study showed that the insulation of HABR decreased PO_4_^3-^ removal by 7%[45], where the both HABRs were operated for 50 d (40 d for 30 h HRT, and 10 d for 20 h HRT). It was found that the PO_4_^3-^ removal was ceased late after 35 d in HABR (I) (instead 20 d in HABR (U)) because of biological phosphorus release by fermentative bacteria by producing fatty acids resulting higher phosphate concentration in the effluent. As a consequence, it decreased PO_4_^3-^ removal efficiency, which concerned with the finding reported by Schön et al. [47]. However, once HABR (I) recovered this situation and ran for an addition 100 d at 20 h HRT (except system idle for 42 days), higher PO_4_^3-^ removal 50±22% was achieved by HABR (I) compare to 40±23% in HABR (U). It indicated that the insulation of HABR also increased PO_4_^3-^ removal efficiency when operated for a longer time (140 d), which opposed to recent findings operating for a shorter time (e.g., 50 d)[45].

### Microalgae cultivation, harvesting, lipid extraction, and biofuels conversion

#### Microalgae cultivation and biomass growth

**Fig 5:**
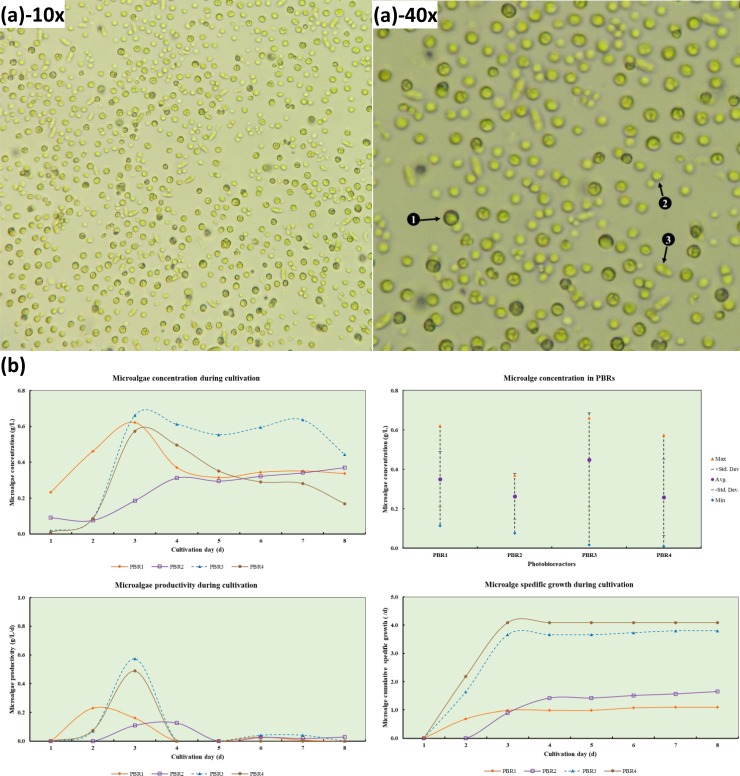
Microalgae species as observed under the light microscope and monitoring data during cultivation. **(a)** Co-culture microalgae species, as seen on day 6 (10x –left, 40x-right (1—*Chlorella vulgaris*, 2—*Chlorella sorokiniana*, *and* 3—*Scenedesmus simris002*)). **(b)** Microalgae concentration during cultivation and within PBRs; and productivity and specific growth rate during eight (8) days of cultivation.

Fig 5(A) shows the co-culture of *Chlorella vulgaris*, *Chlorella sorokiniana*, *and Scenedesmus simris002*, which was cultivated in four PBRs (PBR-1 to PBR-4) for eight days (S3 Table). During microalgae cultivation pH, DO, ORP and EC on a daily basis; and light irradiation and culture media temperature in morning (e.g., AM), noon, and afternoon (e.g., PM) were monitored as presented in Table 3. The average pH was 9.5±0.2 in PBR-1, 9.6±0.3 in PBR-2, 9.6±0.4 in PBR-3 and 9.7±0.4 in PBR during eight days of cultivation. The airflow to supply CO_2_ in PBRs was 10 min/hr continuously during the entire cultivation period. The average pH of the effluent from HABR (U) was 8.0±0.2 raised in PBRs due to air sparging. As reported by Hu et al.[48] that high pH (8.5–10) resulted in intense chemical precipitation of PO_4_^3-^, and also decreased the bioavailability of inorganic carbon resulting lower NO_3_^-^-N removal from wastewater. They suggested that the problem could be resolved by controlling pH or CO_2_ sparging. In this study, it could be resolved by only air sparging during day time (during light irradiation) instead of 24/7. The DO, EC and ORP results suggested favorable culture media existed throughout the cultivation period.

**Table 3:** Monitoring parameters of culture media during microalgae cultivation in PBRs.

Parameter	Unit	Photobioreactors			
		PBR-1	PBR-2	PBR-3	PBR-4
pH	-	9.5±0.2	9.6±0.3	9.6±0.4	9.7±0.4
DO	mg/L	6.3±0.9	6.4±0.7	6.4±0.9	6.4±1.0
ORP	mV	155±61	133±43	124±43	113±37
EC	μS/cm	1749±67	1732±45	1702±86	1751±51
Light (AM)	μmol/m^2^/s	881±393	33±5	110±31	106±20
Light (Noon)	μmol/m^2^/s	110±23	23±7	644±485	107±33
Light (PM)	μmol/m^2^/s	18±11	6±8	38±63	25±35
Media Temp (AM)	(°C)	37±3	34±2	35±2	35±2
Media Temp (Noon)	(°C)	38±3	37±3	39±4	38±3
Media Temp (PM)	(°C)	36±3	36±3	38±5	37±4

**Fig 6:**
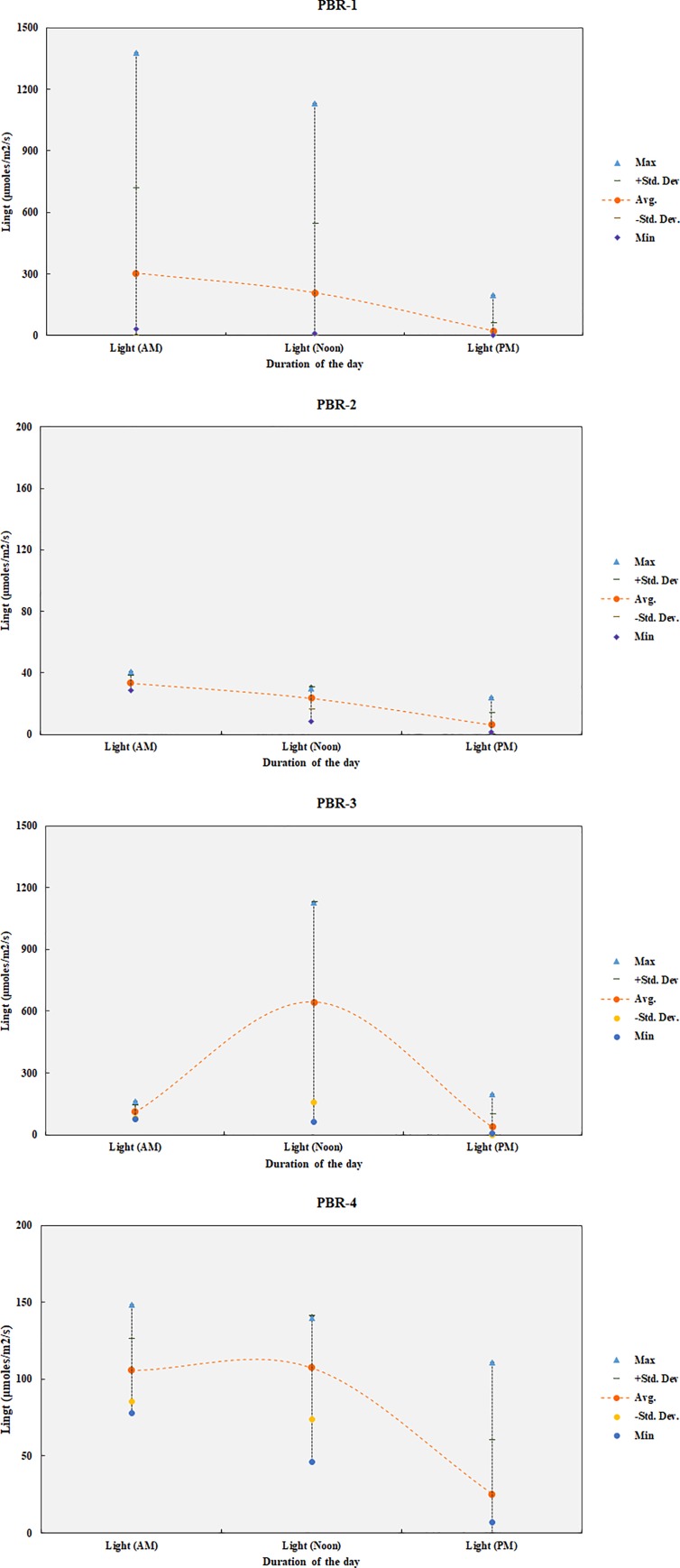
Light irradiation pattern during microalgae cultivation on PBRs (1–4).

The light irradiation during the morning (e.g., AM), noon and afternoon (e.g., PM) were monitored as presented in Table 3. The microalgae concentration (g/L), productivity (g/L/d), specific growth rate (/d) during cultivation are presented in Table 4 and shown in Fig 5(B). The irradiation patterns on PBRs during cultivation are also shown in Fig 6 to observe the effect of light irradiation pattern on algal growth.

**Table 4:** Analysis of microalgae growth in PBRs during cultivation for eight days.

Parameters	Units	Photobioreactors			
		PBR1	PBR2	PBR3	PBR4
μ_avg_	(/d)	0.14	0.21	0.48	0.51
μ_max_	(/d)	0.69	0.90	2.02	2.18
Day of μ_exp_	(d)	1–3	2–4	2–3	2–3
t_d_ (μ_avg_)	(d)	5	3	1	1
X_max_	(g/L)	0.62	0.37	0.66	0.57
P_max_	(g/L/d)	0.23	0.13	0.57	0.49
$P_{{CO}_{2}}$ (avg)	(g/L/d)	0.10	0.07	0.17	0.13

The results show that similar microalgal concentration in most PBRs except PBR-2 (e.g. low X_max =_ 0.37 g/L). This was perhaps due to low light irradiation in PBR-2 (morning: 33±5 μmol/m^2^/s, noon: 23±7 μmol/m^2^/s, afternoon: 6±8 μmol/m^2^/s, respectively) compare to other PBRs (Table 4). The microalgal productivity (e.g. P_max_) were observed high in PBR-3 (0.57 g/L/d) and PBR-4 (0.49 g/L/d) compare to PBR-1 (0.23 g/L/d) and PBR-2 (0.13 g/L/d). Both in PBR-1 and PBR-2, the light irradiation was high in the morning which then gradually decreased at noon and in the afternoon; however, in PBR-3 it was low in the morning then significantly increased at noon and decreased in the afternoon; and in PBR-4 it was mostly similar during the morning and noon but decreased in the afternoon.

The highest productivity 0.57 g/L/d in PBR-3 was perhaps due to this light irradiation pattern, which was low in the morning (110±31 μmol/m^2^/s) then gradually raised at noon (644±485 μmol/m^2^/s) and then dropped in the afternoon (38±63 μmol/m^2^/s). This suggested that the light irradiation pattern had a significant impact on microalgal growth. This indicates that the maximum microalgal growth could be achieved if the light irradiation pattern is similar to PBR-3 (e.g., low at the beginning, then gradually raises and then drops).

#### Lipid content

As mentioned above, two (2) concentrated wet microalgal samples were used for lipid extraction using the single-step procedure for lipid content (LP1 and LP2) by gravimetrical determination[36]. The lipid content of sample LP1 was 44.1%, and LP2 was 38.0%. The relative percent difference (% RPD) for samples LP1 and LP2 was 14.8%, was considered to be acceptable[49,50]. Jena et al.[29] reported 15.5% lipid content from *Chlorella sp*., and 24.0% from *Scenedesmus sp*. *in* autotrophic microalgal cultivation. Also, Ravindran et al.[30] also reported 29.3–56.7% lipid from *Chlorella sp*. and 16–40% lipid from *Scenedesmus sp.*. cultivation. This study also suggests the co-culture of *Chlorella vulgaris*, *Chlorella sorokiniana*, *and Scenedesmus simris002* also yielded high lipid content (38–44.1%) while cultivating from the effluent of HABR (U).

#### Fourier transform infrared spectroscopy (FTIR) analysis

**Fig 7:**
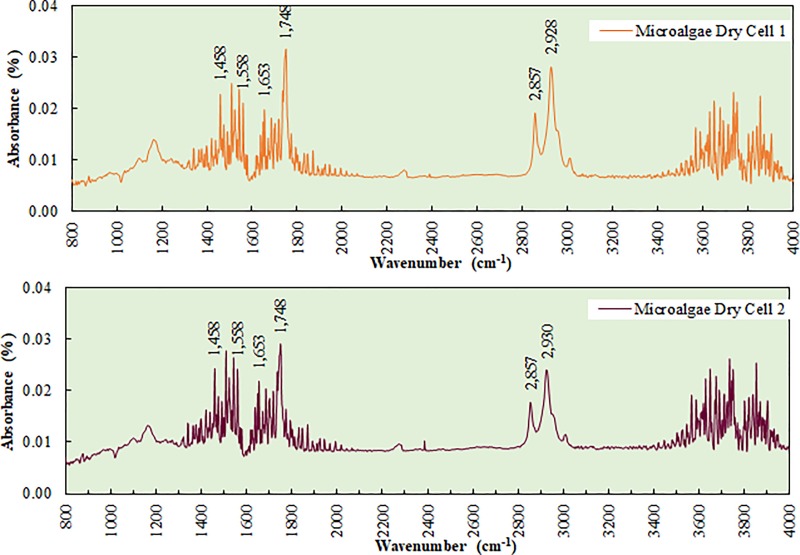
FTIR absorbance spectrum on microalgae dry cell samples.

**Fig 8:**
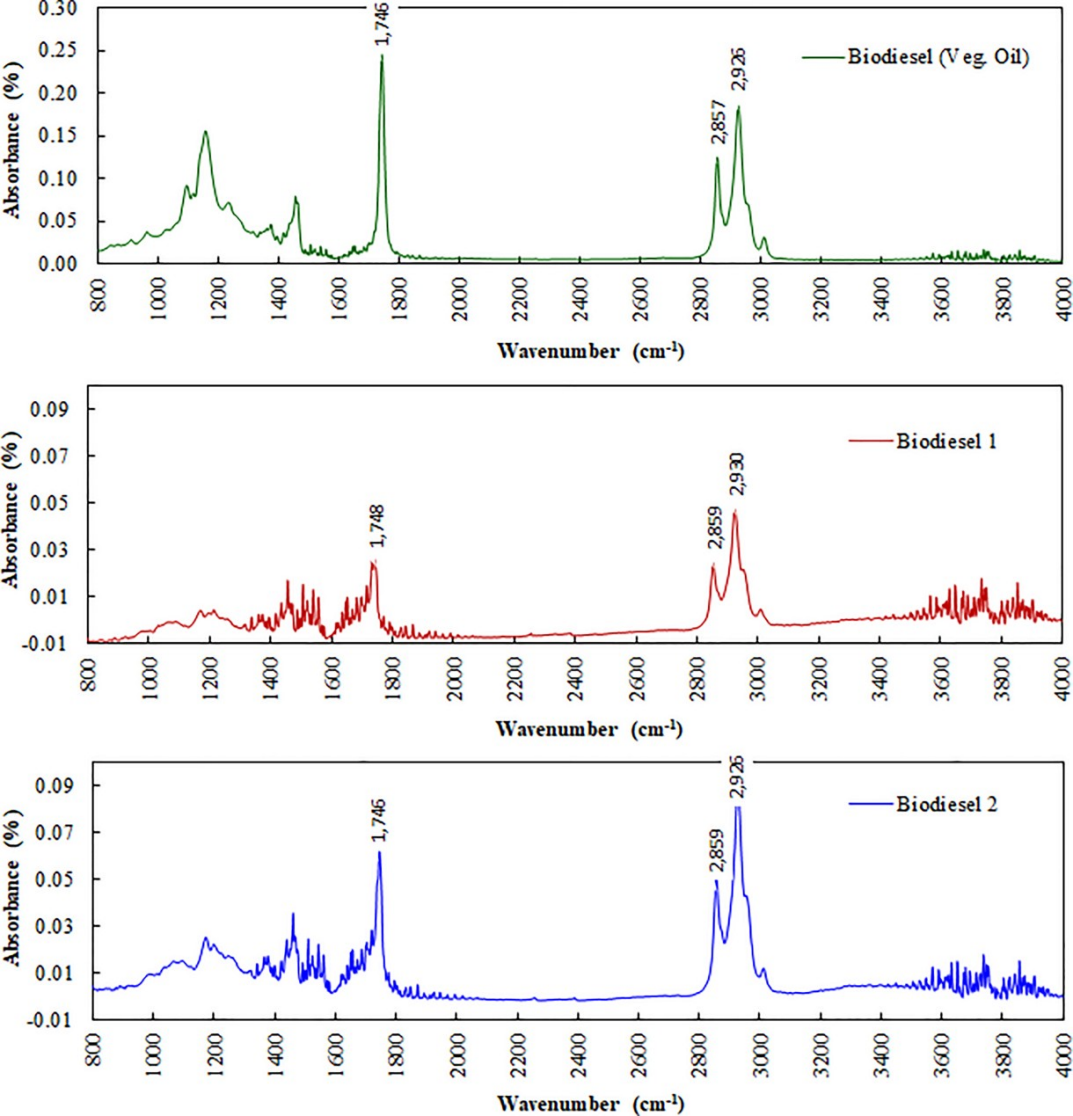
FTIR absorbance spectrum on biofuels samples after transesterification.

FTIR Spectroscopy was employed to determine the presence of vibrations (stretching and bending) active functional groups (including -CH_3_, (CH_2_)_4_-C, C = O, C-O, CH, COO and C-O-C) in the microalgae dry cell (1 & 2), biodiesel (veg. oil) and biodiesel (1 & 2) samples as presented in Table 5 (S4 File) [34]. The most characteristic IR spectra peaks of the microalgae dry cell samples (1and 2) are shown in Fig 7, and biodiesel (veg. oil) and biodiesel (1 & 2) samples are shown in Fig 8. IR spectra for all samples were found to be contaminated for CO_2_ contamination (650–700 cm^-1^ and 2250–2450 cm^-1^) and water vapor ((1300–2000 cm^-1^ and 3440–3950 cm^-1^). After removing the contamination for CO_2_, water vapor, and baseline correction, they were analyzed for active functional groups.

**Table 5:** FTIR band assignments for microalgae dry cell and biodiesel samples.

**Functional groups**	**Spectra range (cm**^**-1**^**)**	**Strength of Spectra ranges**		
		**Microalgae dry cell 1**		**Microalgae dry cell 2**
-CH_3_ stretch	2920–2930	Medium		Medium
	2860–2870	Medium		Medium
C-C-COOH (C = O)	1690–1715	Strong		Strong (1650–1670)
	1700–1725	Strong		-
	1720–1740	-		-
C-CX-COOH (C-O)	1211–1320	Strong		Strong
C = C-COOH (O-H)	875–960	Medium		Medium
	1395–1440	Weak		Weak
	2900–3100	Medium		Medium
CH (Aromatics)	860–900	Strong deformation (bending)		Strong deformation (bending)
**Functional groups**	**Spectra range (cm**^**-1**^**)**	**Strength of Spectra ranges**		
		**Biodiesel** **(Veg. Oil)**	**Biodiesel 1**	**Biodiesel 2**
(CH_2_)_4_-C	2916–2936	Strong antisymmetric	Strong antisymmetric	Strong antisymmetric
	2843–2863	Strong	Strong	Strong
	1445–1485	Medium	Medium	Medium
-CH_3_	2920–2930	Medium	Medium	Medium
	2860–2870	Medium	Medium	Medium
C = O	1712–1720	-	Strong	Strong (1625–1660)
COO (Esters)	17720–1770	-	-	Strong
C-O-C	1000–1300	-	-	Medium
C-O	1130–1070	Strong	-	-

Note: IR spectra for all samples were corrected for CO_2_ contamination (650–700 cm^-1^ and 2250–2450 cm^-1^) and water vapour ((1300–2000 cm^-1^ and 3440–3950 cm^-1^). In addition, biodiesel (1 &2) were corrected for baseline.

Both microalgae dry cells (1 & 2) samples were found to match more than 95% with Crisco cooking, sesame, or olive oil. Also, both samples were shown similar strength of function group peaks (Table 5). The strong absorbance peaks were observed for C-O (1211–1320 cm^-1^) and C = O (1690–1715 cm^-1^ and 1700–1725 cm^-1^) functional groups. The medium strength peaks were also observed for–CH_3_ stretch (2860–2870 cm^-1^ and 2920–2930 cm^-1^), O-H (875–960 cm^-1^, 1395–1440 cm^-1^ and 2900–3100 cm^-1^). The results suggested that these samples had high lipid contents for biodiesel conversion.

The biodiesel (veg. oil) and biodiesel (1 & 2) samples were found more than 95% matching with methyl ester when IR data were corrected for CO_2_, water vapor, and baseline. All samples showed similar strength on corresponding functional groups (Fig 8). There was medium to strong strength peaks observed for (CH_2_)_4_-C (1445–1485 cm^-1^, 2843–2863 cm^-1^ and 2916–2936 cm^-1^) and–CH3 stretch (2860–2870 cm^-1^ and 2920–2930 cm^-1^). Also, biodiesel 1 showed a strong peak for C = O (1712–1720 cm^-1^), biodiesel 2 showed a strong peak for COO (ester, 1720–1770 cm^-1^), and biodiesel (veg. oil) showed a strong peak for C-O (1070–1130 cm^-1^). The results indicate the successful transesterification conversion of the microalgal dry cell lipid to biodiesel [34,40,51].

#### Gas chromatography-flame ionization detector (GC-FID) analysis

**Fig 9:**
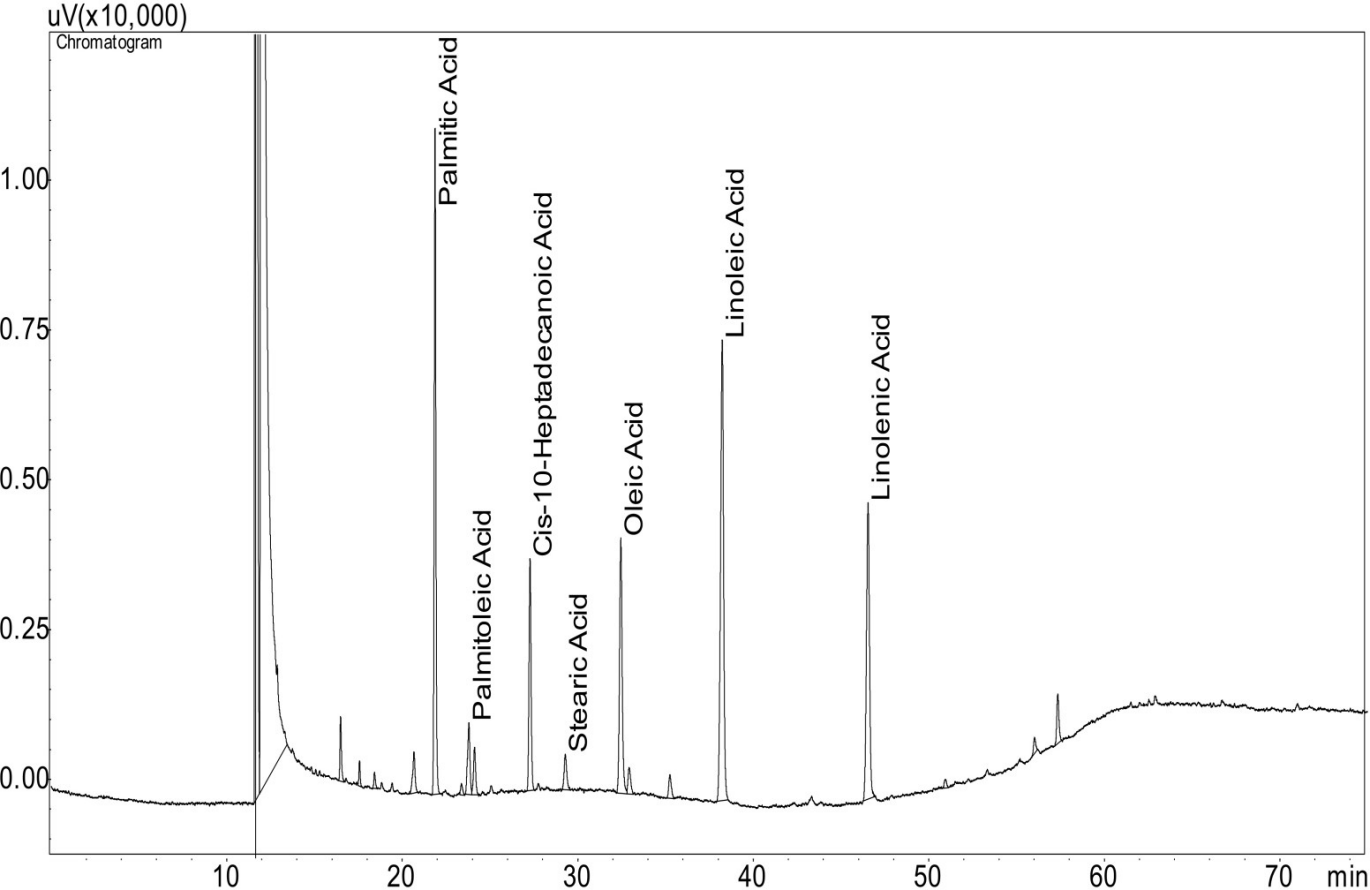
GC-FID chromatogram of FAMEs composition of microalgal biodiesel.

**Fig 10:**
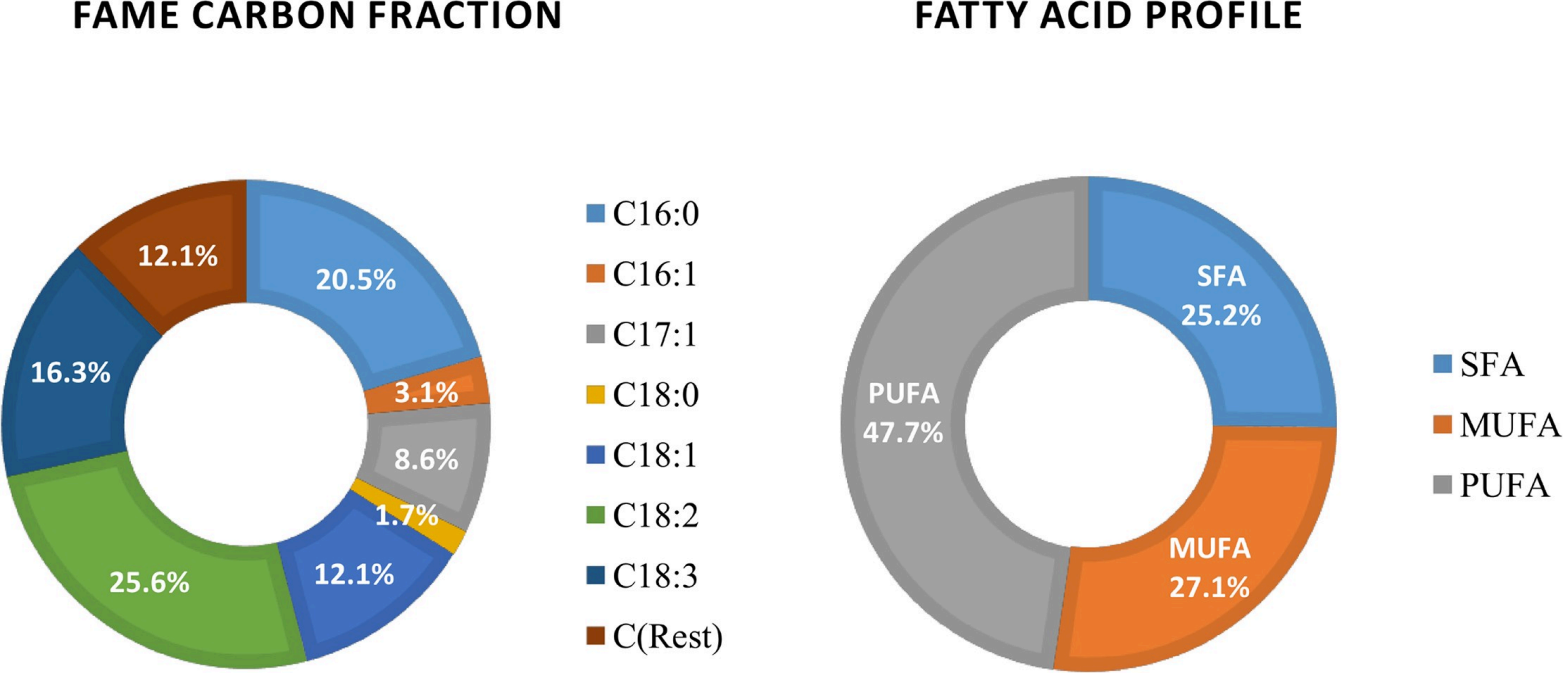
FAME Carbon (C16-C18) fraction and fatty acid profile of microalgal biodiesel.

The results of the GC-FID analysis of biodiesel sample (biodiesel 2) are presented in Fig 9, which shows that the major components of biodiesel consist of methyl esters of palmitic (C16:0), palmitoleic (C16.1), heptadecanoic (C17:1), stearic (C18:0), oleic (C18:1), linoleic (C18:2) and linolenic (C18:3) acids. The FAME profile was similar to the finding reported by several researchers [36,39,40,52]. In addition, the dominant FAME components were palmitic acid (C16:0) of 20.5%, heptadecanoic acid (C17:1) of 8.6%, oleic acid (C18:1) of 12.1%, linoleic acid (C18:2) of 25.6%, and linolenic acid (C18:3) of 16.3% (Fig 10). This indicates that the major FAME components (87.9%) of the microalgal biodiesel lies between C16 and C18, which provides several advantages: quality ignition, low viscosity resulting lubricity, and higher oxidative stability for longer storage[39,40,52–54]. This FAMEs content 87.9% is higher than 86.41% (microwave irradiation for lipid extraction) as reported by Wahidin et al.[52] in their study. In particular, the FAME profile was similar to FAMEs in biodiesel extracted from soybean, canola, and palm (e.g., palmitic acid, stearic acid, oleic acid, linoleic acid, and linolenic acid) [39].

Fig 10 shows the major FAMEs composition contained saturated fatty acid (SFA) of 25.2%, monounsaturated fatty acid (MUFA) of 27.1% and polyunsaturated fatty acid (PUFA) of 47.7%, which was very similar to biofuels from soybean oil (SFA 15.3%, MUFA 25.6%, PUFA 59.1%)[39]. Jena et al.[29] reported biofuels from *Chlorella sp*. had 34% SFA and 66% PUFA, and biofuels from *Scenedesmus sp* culture had 36.5% SFA and 63.5% PUFA. Besides, Ngangkham et al.[55] reported biofuels from *Chlorella sorokiniana* contained 31.8% SFA, 8% MUFA and 60.2% PUFA. In this study, a co-culture of three microalgae (*Chlorella vulgaris*, *Chlorella sorokiniana*, *Scenedesmus simris002*) showed balanced between SFA, MUFA, and PUFA.

#### Wastewater quality after microalgae harvesting

After harvesting microalgal biomass, wastewater in PBRs was analyzed for pH, ORP, turbidity, COD, NO_3_^-^-N, NO_3_^-^-N, PO_4_^3-^ parameters as presented in Table 6. As mentioned above, the average pH was found higher (9.1±0.3) than the pH of HABR (U) effluent (8.0±0.2) due to air sparging. This high pH resulted in significant chemical precipitation of PO_4_^3-^ (from 29.3±18.0 to 0.7±0.2), which also decreased the bioavailability of inorganic carbon resulting lower NO_3_^-^-N removal (12.5±1.3 mg/L) from HABR (U) effluent (24.7±34.5 mg/L)[48]. This situation could be improved by only air sparging during day time (during sunlight irradiation) instead of 24/7. Also, COD concentration was found higher (102±21 mg/L) than HABR (U) effluent (39±37 mg/L), which was perhaps due to microalgal biomass contribution to COD value.

**Table 6:** Characteristics of wastewater before and after harvesting of microalgae in PBRs.

Parameter	Unit	wastewater concentration	
		HABR (U) (before cultivation)	PBRs (after harvesting)
pH	-	8.0±0.2	9.1±0.3
ORP	mV	101.4±75.6	158±16
Turbidity	NTU	11±7	41±7
COD	mg/L	39±37	102±21
NH_4_^+^-N	mg/L	37.8±28.0	-
NO_3_^-^-N	mg/L	24.7±34.5	12.5±1.3
NO_2_^-^-N	mg/L	12.1±22.7	9.5±2.4
PO_4_^3-^	mg/L	29.3±18.0	0.7±0.2

### The overall performance of HABR-PBR system

The study suggests that both HABRs were capable of removing most of the organic (COD removal of 93±7% by HABR (U), and 89±9% by HABR (I)) and suspended solid (TSS removal of 91±9% by HABR(U), and 96±5% by HABR (I)) from domestic wastewater. Also, the effluent from HABR (U) contained higher nutrients (high N and P), and lower TC/FC (e.g., 75% TC and 59% FC removal in HABR (U)) than HABR (I), which reduced the risk of bacterial contamination during microalgae cultivation. Hence, the effluent of HABR (U) was considered a healthy feedstock (high N: P = 3:1)[32,33] and was used for microalgae cultivation.

A co-culture of *Chlorella vulgaris*, *Chlorella sorokiniana*, and *Scenedesmus simris002* showed high lipid content up to 44.1%. The study also suggests that the sunlight irradiation pattern also has a significant influence on the productivity of algal biomass. The maximum microalgal growth could be achieved if the light irradiation pattern is similar to PBR-3 (e.g., low at the beginning then gradually raises and then drops). The results of the GC-FID analysis showed that the FAME profile was similar to the finding reported by several researchers [36,39,40,52]: dominant of palmitic acid (C16:0) of 20.5%, heptadecanoic acid (C17:1) of 8.6%, oleic acid (C18:1) of 12.1%, linoleic acid (C18:2) of 25.6%, and linolenic acid (C18:3) of 16.3%, which resulted 87.9% of the major FAME components (C16-C18). The quality of biodiesel would provide several advantages: quality ignition, low viscosity resulting lubricity, and higher oxidative stability for longer storage[39,40,52–54].

## Conclusion

In this research, a simplistic sustainable approach of algal biofuels production from wastewater was proposed using the HABR-PBR system. The study suggests that the HABR was capable of removing most of the organic and solid (>90% COD and TSS removal) from wastewater, and produced a healthy feedstock (high N: P = 3:1) for microalgae cultivation in PBRs for biofuels production. A co-culture of *Chlorella vulgaris*, *Chlorella sorokiniana*, and *Scenedesmus simris002* showed high lipid content up to 44.1%; and higher 87.9% of dominant FAMEs composition (C16-C18) in biodiesel.

The results suggest significant quality improvement of wastewater after microalgae cultivation and harvesting; however, there is still addition scopes to improve this HABR-PBR system, perhaps by reducing air sparging during cultivating, post flocculation of algal biomass before discharge. The study also suggests that there is a potential opportunity to re-use this treated water with further improvement to reduce water footprint for these developing countries. The HABR-PBR technological approach (i.e., a problem turned into a solution and resource) will work as a double-edged solution to mitigating wastewater problems and cogenerating algal biomass to produce bioenergy (as biofuels) to overcome energy demand especially for those countries located in a subtropical/tropical region with a warm climate.

## Supporting information

S1 Table pH data of both HABR (U) and HABR (I). (PDF)

S2 Table ORP data of both HABR (U) and HABR (I). (PDF)

S3 Table Microalgae concentration (as Abs), temperature and light irradiation data during eight (8) days of cultivation. (PDF)

S1 File ANOVA analysis of Loading Rate (LR) and Removal Rate (RR) of both Ammonia-N and Nitrate-N for both reactors (HABR (U) and HABR (I)). (XLSX)

S2 File Temperature data of both HABR (U) and HABR (I). (XLSX)

S3 File NH_4_^+^-N, NO_3_^-^-N, and PO_4_^3^- data of HABR (U) and HABR (I). (XLSX)

S4 File FTIR data for microalgae dry cells and biodiesel samples. (XLSX)
