# Supplementary material for: A simplistic approach of algal biofuels production from wastewater using a Hybrid Anaerobic Baffled Reactor and Photobioreactor (HABR-PBR) System
Source: PLoS One. 2019 Dec 5;14(12):e0225458. doi: 10.1371/journal.pone.0225458 (PMC6894839; doi:10.1371/journal.pone.0225458)
Supplement: S1 Table — (PDF) [file pone.0225458.s001.pdf]

S1 Table. pH data of both HABR (U) and HABR (I).

**Day**	***U-R***	***U-1***	***U-2***	***U-3***	***U-4***	***U-5***	***U-6***	***U-7***	***U-E***	**I-R**	**I-1**	**I-2**	**I-3**	**I-4**	**I-5**	**I-6**	**I-7**	**I-E**
**7**	8.03	8.01	8.03	8.01	7.99	7.99	7.96	8.1	8.14	8.03	7.99	7.99	7.96	7.96	7.95	7.93	7.97	7.92
**14**	8.11	8.14	8.15	8.16	8.16	8.18	8.17	8.22	8.3	8.12	8.12	8.15	8.15	8.14	8.09	8.11	8.08	8.09
**18**	8.04	8.03	8.03	8	7.9	7.95	7.95	7.94	7.94	8.08	8.07	8.07	8.01	7.84	7.82	7.83	7.8	7.83
**27**	8.22	8.16	8.13	8.08	8.05	8.03	7.99	7.97	8.01	8.24	8.22	8.22	8.2	8.19	8.27	8.15	8.17	8.14
**29**	8.25	8.16	8.16	8.11	8.09	8.04	8.02	8	8.03	8.25	8.21	8.21	8.2	8.19	8.18	8.19	8.16	8.18
**35**	7.94	7.93	7.92	7.92	7.92	7.92	7.9	7.91	7.95	7.94	7.91	7.92	7.92	7.93	7.92	7.97	7.97	8
**45**	7.74	7.75	7.76	7.68	7.66	7.62	7.57	7.68	7.8	7.72	7.72	7.72	7.69	7.71	7.75	7.8	7.76	7.82
**50**	7.97	7.82	7.97	7.94	7.91	7.86	7.77	7.8	7.81	7.98	7.77	7.97	7.96	7.95	7.93	7.97	7.97	7.98
**57**	8.1	8.19	8.09	8.09	8.13	8.14	8.11	8.24	8.34	8.12	8.14	8.12	8.12	8.13	8.14	8.16	8.17	8.19
**108**	8.12	7.99	7.99	7.99	7.92	7.93	7.86	7.85	7.89	8.12	7.92	8.02	7.99	8.04	8.05	8.04	8.05	8
**113**	8.32	8.22	8.2	8.16	8.13	8.1	8.09	8.14	8.2	8.35	8.2	8.23	8.23	8.2	8.2	8.2	8.19	8.19
**120**	7.98	7.88	7.93	7.87	7.85	7.86	7.83	7.76	7.82	7.91	7.98	7.88	7.91	7.9	7.82	7.92	7.92	7.89
**127**	8.21	7.86	7.97	7.95	7.95	7.94	7.91	7.9	7.83	8.18	7.87	8.09	8.06	8.03	8	8	7.98	7.99
**139**	7.94	7.87	7.95	7.9	7.88	7.89	7.95	7.85	7.88	7.94	7.9	7.98	7.98	7.95	7.92	7.99	7.98	7.9
