# Supplementary material for: A simplistic approach of algal biofuels production from wastewater using a Hybrid Anaerobic Baffled Reactor and Photobioreactor (HABR-PBR) System
Source: PLoS One. 2019 Dec 5;14(12):e0225458. doi: 10.1371/journal.pone.0225458 (PMC6894839; doi:10.1371/journal.pone.0225458)
Supplement: S2 Table — (PDF) [file pone.0225458.s002.pdf]

S2 Table. ORP data of both HABR (U) and HABR (I).

Day	*U-R*	*U-1*	*U-2*	*U-3*	*U-4*	*U-5*	*U-6*	*U-7*	*U-E*	I-R	I-1	I-2	I-3	I-4	I-5	I-6	I-7	I-E
**22**	49	50	51	58	53	54	49	50	51	49	50	51	52	50	51	54	49	48
**27**	93	94	92	91	92	95	96	94	98	112	114	104	106	104	101	92	91	95
**29**	63	64	64	64	64	64	65	64	63	57	56	57	58	59	60	60	62	62
**35**	86	84	82	89	88	87	88	88	87	89	75	77	79	80	80	80	81	82
**45**	91	87	92	95	86	84	81	75	81	99	98	97	98	100	97	95	94	95
**50**	-60	-315	-14	173	202	210	216	234	255	-30	-130	11	11	7	7	6	-6	-12
**50**	-60	-315	-14	173	202	210	216	234	255	-30	-130	11	11	7	7	6	-6	-12
**57**	230	110	105	110	112	114	115	114	113	99	95	116	117	117	116	116	117	115
**108**	4	-13	-5	-6	32	26	22	50	85	-46	-137	-58	-77	-49	-33	-2	11	45
**113**	56	62	48	45	44	38	65	57	85	52	56	54	52	48	52	65	58	72
**120**	-80	-185	23	22	20	17	16	14	11	-10	-3	-16	-10	-6	-2	-1	5	10
**127**	-10	-26	30	34	31	27	24	20	14	1	-8	25	24	23	21	19	18	19
**139**	112	-50	32	36	28	26	22	16	120	110	-5	54	32	26	25	35	78	124
