# Supplementary material for: A simplistic approach of algal biofuels production from wastewater using a Hybrid Anaerobic Baffled Reactor and Photobioreactor (HABR-PBR) System
Source: PLoS One. 2019 Dec 5;14(12):e0225458. doi: 10.1371/journal.pone.0225458 (PMC6894839; doi:10.1371/journal.pone.0225458)
Supplement: S3 Table — (PDF) [file pone.0225458.s003.pdf]

S3 Table. Microalgae concentration (as Abs), temperature and light irradiation data during eight (8) days of cultivation.

Sample ID	Day	Absorbance	AM Light (µmoles/m2/s)	Noon Light (µmoles/m2/s)	PM Light (µmoles/m2/s)	Average Light	Avg. AM Media Temp (°C)	Avg. Noon Media Temp (°C)	Avg. PM Media Temp (°C)
**S1**	1	0.104	270	70	19	120	31.5	30.8	29.8
**S2**	1	0.046	31	9	9	16	30.5	30.8	29.8
**S3**	1	0.016	138	61	20	73	31.5	30.8	29.8
**S4**	1	0.013	99	46	15	53	31.5	30.8	29.8
**S1**	2	0.198	472	114	13	200	34.0	35.5	33.5
**S2**	2	0.040	41	16	3	20	33.5	34.5	33.3
**S3**	2	0.045	161	88	18	89	34.0	35.0	33.8
**S4**	2	0.044	148	65	15	76	34.0	35.5	33.5
**S1**	3	0.264	602	112	9	241	37.0	36.5	35.0
**S2**	3	0.085	32	27	1	20	34.5	36.5	34.5
**S3**	3	0.280	96	92	9	65	35.0	36.5	34.8
**S4**	3	0.244	106	122	7	78	35.0	37.5	34.8
**S1**	4	0.161	1,167	101	44	437	37.5	39.0	37.5
**S2**	4	0.137	41	28	24	31	35.0	38.5	38.5
**S3**	4	0.260	97	1,111	194	468	36.5	41.0	42.5
**S4**	4	0.212	108	112	111	110	36.5	39.5	40.5
**S1**	5	0.138	1,376	151	12	513	40.5	40.5	38.0
**S2**	5	0.130	31	30	3	21	35.5	39.5	39.0
**S3**	5	0.236	75	1,130	15	407	36.0	41.5	42.0
**S4**	5	0.153	78	123	12	71	38.5	40.0	40.0
**S1**	6	0.150	981	111	13	369	39.5	40.0	37.0
**S2**	6	0.141	31	27	3	20	36.5	39.0	36.0
**S3**	6	0.446	139	741	11	297	37.5	42.5	40.5
**S4**	6	0.128	111	130	10	84	37.5	41.0	38.5
**S1**	7	0.153	1,231	97	19	449	38.5	39.0	36.5
**S2**	7	0.149	31	25	4	20	34.5	38.5	37.5
**S3**	7	0.270	81	1,074	20	392	34.5	41.5	41.0
**S4**	7	0.124	91	122	16	77	34.5	39.0	38.5
**S1**	8	0.147	946	122	19	362	38.5	40.5	38.0
**S2**	8	0.161	29	27	4	20	35.5	39.0	38.5
**S3**	8	0.191	96	859	18	325	36.0	43.0	41.0
**S4**	8	0.078	106	140	15	87	35.5	40.3	39.0
